# RNA Deregulation in Amyotrophic Lateral Sclerosis: The Noncoding Perspective

**DOI:** 10.3390/ijms221910285

**Published:** 2021-09-24

**Authors:** Pietro Laneve, Paolo Tollis, Elisa Caffarelli

**Affiliations:** 1 Institute of Molecular Biology and Pathology, National Research Council, 00185 Rome, Italy; 2 Department of Biology and Biotechnology Charles Darwin, Sapienza University of Rome, 00185 Rome, Italy; paolo.tollis@uniroma1.it

**Keywords:** ALS, motoneurons, neurodegeneration, RNA metabolism, noncoding RNAs, microRNAs, long noncoding RNAs, circular RNAs

## Abstract

RNA metabolism is central to cellular physiopathology. Almost all the molecular pathways underpinning biological processes are affected by the events governing the RNA life cycle, ranging from transcription to degradation. The deregulation of these processes contributes to the onset and progression of human diseases. In recent decades, considerable efforts have been devoted to the characterization of noncoding RNAs (ncRNAs) and to the study of their role in the homeostasis of the nervous system (NS), where they are highly enriched. Acting as major regulators of gene expression, ncRNAs orchestrate all the steps of the differentiation programs, participate in the mechanisms underlying neural functions, and are crucially implicated in the development of neuronal pathologies, among which are neurodegenerative diseases. This review aims to explore the link between ncRNA dysregulation and amyotrophic lateral sclerosis (ALS), the most frequent motoneuron (MN) disorder in adults. Notably, defective RNA metabolism is known to be largely associated with this pathology, which is often regarded as an RNA disease. We also discuss the potential role that these transcripts may play as diagnostic biomarkers and therapeutic targets.

## 1. Introduction

Amyotrophic lateral sclerosis (ALS) is an aging-related and lethal neurodegenerative disorder characterized by the progressive degeneration of motoneurons (MNs) in the spinal cord (SC), brainstem (BS), and motor cortex (MCx). The consequent motor axonal retraction causes muscle weakness and progressive paralysis as major symptoms. Death usually occurs due to respiratory failure, generally within three to five years of onset. However, population-based studies revealed that ALS involves the central nervous system (CNS) more extensively than previously imagined. In particular, up to 50% of ALS patients develop cognitive and behavioral alterations and about 13% have concomitant frontotemporal dementia (FTD), which led to considering ALS and FTD as the two ends of one clinicopathological spectrum [1,2]. 

ALS has been classified into familial ALS (fALS), representing about 10% of cases, and sporadic ALS (sALS). They are indistinguishable from a clinical point of view, except for the onset which is earlier in fALS [3]. The latter can be inherited in an autosomal dominant manner and, more rarely, in an autosomal recessive or X-linked manner [4,5]. Cases of fALS have been attributed to mutations, mostly missense substitutions, in more than 20 genes. Among these, four genes, namely *SOD1* (Cu-Zn superoxide dismutase 1), *C9ORF72* (hexanucleotide expansion repeat in chromosome 9 open reading frame 72), *TARDBP* (transactive response DNA-binding protein 43 kDa), and *FUS* (fused in sarcoma), account for up to 70% of all cases of fALS [5,6,7]. Individuals who do not have affected relatives are classified as sALS patients. 

DNA sequencing analyses carried out in patients with sALS revealed that 1–3% of cases are caused by *SOD1* mutations [8] and 5% by intronic expansion in *C9ORF72* [9]. Mutations in the other ALS-associated genes, such as *TARDBP*, coding for TDP-43 protein, *FUS*, *HNRNPA1*, *SQSTM1*, *VCP*, *OPTN*, and *PFN1*, are rare in sALS, whose onset could be contributed to by environmental factors [10].

ALS was initially interpreted as a proteostasis failure [10]. This view was supported by the finding that some mutated RNA-binding proteins (RBPs), such as the components of the ribonucleoprotein (RNP) granules TDP-43 and FUS, are delocalized in the cytoplasm where they form pathological aggregates [11]. This phenomenon is exacerbated by the alterations of the two main pathways of protein clearance, namely the ubiquitin–proteasome system [12] and autophagy [13]. However, the same ALS-associated proteins are regulators of RNA metabolism, leading to a further interpretation of the pathology as an RNA disorder. Interacting with thousands of RNA targets, they affect splicing, transport, stability, and even translation, which means that a disturbance in the function of these proteins may affect RNA metabolism on a broad scale [14]. As an example, cross-linking immunoprecipitation (CLIP)-Seq analysis unveiled more than 39,000 TDP-43-binding sites in the mouse transcriptome [15]. Furthermore, the splicing patterns of 965 messenger RNAs (mRNAs), whose products were mainly involved in synaptic activity, were altered upon reduction of the protein from adult mouse brain, Indicating that TDP-43 is key to normal splicing patterns of several brain-enriched mRNAs [15,16]. Similarly, alternative splicing of mRNAs was altered in *FUS*-related ALS, with consequent deregulation of neuronal gene expression and production of thousands of aberrantly processed mRNAs [17]. The fact that these ALS-associated proteins intervene not only in the metabolism of mRNAs, with dramatic consequences on protein products, but also in noncoding RNAs (ncRNAs) with an impact on the biological processes they control, is of growing interest. A clear example is the role played by TDP-43 and FUS in the biosynthesis of microRNAs (miRNAs), small ncRNAs that orchestrate differentiation and developmental programs by pleiotropically regulating gene expression [18,19].

Based on these considerations, ALS has also been proposed as an RNA-mediated neuropathology, which better reflects the heterogeneity of the disease [10]. 

Here, we describe the current state of the art about the relevant contribution given by specific classes of ncRNAs to the pathology.

## 2. A Brief History of ALS

ALS is also called Lou Gehrig’s disease in the United States and MN disease in the United Kingdom [10]. The name of the pathology reflects both the degeneration of the upper MNs, whose axons project from the cortex to the BS and lateral SC (lateral sclerosis), and the death of lower MNs, which project from the BS or SC to the muscle, causing its wasting (amyotrophy). It was first described as a specific entity in 1869 by the neurologist Jean-Martin Charchot [20]. In the mid-1900s, Kurland and Mulder, carrying out a study on a case series of 58 patients, reported 10% familial cases [21,22]. More recently, the combination of population-based epidemiological studies with advanced genetics and the development of new bioinformatics tools and neuroimaging techniques led to considering ALS as a syndrome encompassing a wide clinical and pathological spectrum. These findings prompted further stratification of ALS into subtypes, which will be very helpful for the prediction of prognosis and for the design of specific treatments based on different disease mechanisms.

Different criteria have been used for classifying ALS. The traditional definition of ALS subtypes, based on the involvement of upper or lower MNs, was overtaken by other classifications relying on different parameters. A statistical method was developed that predicts prognosis with more accuracy than do clinical phenotypes. It consisted of applying latent class cluster analysis to a large database including 1467 records of ALS patients. This method provided five phenotypic classes of ALS that strongly predicted survival [23]. Another classification of ALS is based on the site of onset and the involvement of different sets of MNs. Accordingly, four forms can be diagnosed: (i) progressive muscular atrophy, which mainly affects spinal neurons or lower MNs and causes limb weakness and atrophy; (ii) primary lateral sclerosis, which primarily affects corticospinal MNs and causes spasticity with increased limb tone; (iii) bulbar ALS, a devastating variant, that mainly affects BS MNs innervating tongue muscles, causing difficulties in speech, chewing, and swallowing; (iv) pseudobulbar palsy, that affects cortical frontobulbar MNs and causes emotion accentuation, absence of facial expression, spastic dysarthria, and dysphagia [10,24]. To date, none of the used classifications include the cognitive and behavioral symptoms. A range of subtypes should also be highlighted to overcome the heterogeneity of ALS and define subcohorts of patients to address personalized treatments.

## 3. Face with ALS: Onset, Clinical Manifestation, and Diagnosis 

As an aging-related neurodegenerative disease, the occurrence of ALS is growing with the increasing aging of the population [14]. It is the most common adult-onset MN disease diagnosed in 1–2 cases per 100,000 individuals every year in most countries and it is, therefore, considered an orphan disease. However, its inevitably lethal outcome gives incommensurate importance to its occurrence. In the United Kingdom and the United States, ALS determines more than 1 in every 500 deaths in adults, which has led to the prediction that more than 15 million people presently alive across the world will die of the disease [14]. In more detail, population-based studies highlighted that ALS is more common in men than in women [25,26] and that its incidence differs depending on ancestral origin. It is particularly low in the population of mixed ancestral origin in North America (0.63 cases per 100,000 individuals) [27], whereas it is higher in regions with relatively homogeneous populations, such as in European populations (2.6 cases per 100,000 individuals) [28,29].

The age of onset is highly variable but almost always occurs in the fifth or sixth decade of life, at a mean age of 55 years. Presumably, it might begin early in the first two decades of life without clear symptoms and emerge only later during life. Median survival is 2 to 4 years from the onset with only 5–10% of patients surviving longer [30,31]. In particular, many of the long-term survivors show either upper MN or lower MN involvement [32,33]. 

Disease onset begins focally and eventually spreads to other body districts. Patients initially experience muscle weakness, fasciculations, muscle atrophy, spasticity, and hyperreflexia that ultimately lead to paralysis [10]. Astrogliosis and microgliosis, accompanied by mitochondrial dysfunction and defects in axonal transport, are hallmarks of the disorder [10].

The diagnosis of ALS is made difficult by the heterogeneous clinical presentation and the absence of a specific test. It relies on a detailed description of the symptoms, physical examination, electrodiagnostic testing, neuroimaging, and familiar history. The El Escorial or Awaji diagnostic criteria are exploited when there is a history of progressive weakness in one or more body regions and evidence of involvement of upper and lower MNs [34]. Thus far, ALS standard treatment consists of multidisciplinary care, including respiratory support and symptom management, whereas the only U.S. Food and Drug Administration-approved drugs are riluzole and edaverone that have only limited effects on patient survival [35].

The absence of effective treatments for the disease is due to the lack of deeper knowledge of the pathogenic mechanisms responsible for MN death, and to the delayed diagnosis usually made in an advanced pathological state. This could be overcome with the identification of reliable biomarkers for early diagnosis, patient stratification, and for the effectiveness of pharmacological therapies. 

Many studies are going in this direction. They mainly focus on neurofilaments (Nfs), neuron-specific cytoskeletal proteins that are involved in the stabilization and polarization of neural cells and, therefore, in effective axonal conduction. Notably, their concentration increases in biological fluids proportionally to the degree of axonal damage [36].

Although not yet adopted into clinical practice, the levels of phosphorylated neurofilament heavy chain (pNfH) in cerebrospinal fluid (CSF) have been proposed as specific biomarkers for MN disease. pNfH is endowed with the best performance to discriminate between patients with ALS and healthy and neurological controls with a sensitivity of about 90% [37]. Another study explored blood as an alternative source for measuring pNfH levels. ALS patients displayed elevated concentrations of serum pNfH, that correlated with the disease progression rate [38]. However, given the proximity to the degenerating MNs in the brain and SC, CSF pNfH outperformed serum pNfH (10-fold higher than blood) in discriminating ALS patients [36,39]. Recently, single-molecule assays allowed the evaluation of ultralow concentrations of blood Nf, which may be very advantageous since blood samples are easily accessible and attainable in a less invasive way compared to CSF [36].

## 4. RNA Biology of ALS 

The protein-coding genes associated with ALS pathogenesis have been grouped into three main classes: the genes altering proteostasis and protein quality control, those involved in cytoskeletal dynamics, and genes affecting RNA metabolism [10]. Recently, great emphasis has been given to the latter gene class and deregulation of RNA has emerged as a major contributor to ALS. 

Accordingly, the major ALS-causative genes, namely *SOD1*, *C9ORF72*, *TARDBP*, and *FUS*, are involved in the control of RNA metabolism to different degrees. In particular, SOD1 negatively affects the stability and function of some mRNA species by interacting with their 3′-untranslated region (3′-UTR) [40,41,42]. The interaction of mutant SOD1 with vascular endothelial growth factor (*VEGF*) mRNA, besides causing the recruitment of other proteins such as TIAR and HuR into insoluble aggregates, also determines a decrease in *VEGF* mRNA levels. Similarly, as observed in human spinal MN from *SOD1*-ALS cases, the binding of mutant SOD1 to neurofilament light chain (*NFL*) mRNA destabilizes the transcript [40]. The reduction of *NFL* mRNA levels results in an aberrant stoichiometry of NF subunits, NF aggregation, and neurite degeneration in the iPSC-derived model of ALS [42]. Additionally, mutant SOD1 has been shown to induce alternative splicing deregulation [43].

The *C9ORF72* gene could cause ALS through an RNA toxicity mechanism. It carries repeat expansion mutations and accounts for about 50% of fALS and 10% of sALS cases [44]. Both strands of *C9ORF72* repeat expansion are transcribed, producing RNA foci that accumulate in patient tissues [45]. The aberrant RNA foci may, in turn, act as a platform that sequesters several RBPs, such as hnRNP-A3, FUS, and TDP-43, producing alterations in RNA metabolism at a global level [46,47,48]. Accordingly, the use of antisense oligonucleotides (ASOs) targeting *C9ORF72* repeat expansion avoids RNA foci formation and restore the alteration of gene expression in ALS MNs [46,49].

Mutations in the *TARDBP* gene are found in most cases of ALS [50]. Importantly, independent studies carried out in zebrafish [51], *Drosophila* [52,53], cultured mammalian neuronal cells [54,55,56], and mice [57] pointed to the relevance of TDP-43 activity as an RNA processing regulator of neuronal differentiation, synaptic transmission, and neuronal plasticity. Several studies underscored its involvement in every step of RNA metabolism [58] as well as its relevant role in miRNA biosynthesis [55,59,60,61,62].

Mutations in TDP-43 mainly occur in the C terminus, containing the nuclear localization signal, and are responsible for mislocalization of the nuclear protein in the cytoplasm of MNs, where it forms insoluble aggregates. This may cause, at the same time, a loss of function of TDP-43 in the nucleus and a gain of cytoplasmic toxic function, both being detrimental to neuronal function and survival. 

As for TDP-43, FUS is a ubiquitously expressed RBP regulating several aspects of RNA metabolism and processing. It is a predominantly nuclear protein crucially involved in transcription, pre-mRNA splicing, and miRNA biogenesis [63]. However, it shuttles to the cytoplasm [64], particularly in neurons, indicating that it may participate in regulating mRNA transport into neurites and local protein translation at synapses [65,66]. Mutant FUS displays an abnormal cytoplasmic localization in the neurons of ALS patients where it accumulates in cytoplasmic inclusions, the stress granules (SGs) [67,68].

Interestingly, it was demonstrated that the RNA-binding domain of both TDP-43 and FUS is essential for the neurodegenerative phenotype [69]. In particular, it was shown that RNA-binding-incompetent FUS, also carrying ALS-causing mutations, predominantly localizes in the nucleus in both *Drosophila* MNs and in a neuronal cell line [69]. This finding reveals that the aberrant cytoplasmic localization of FUS is mediated by its RNA-binding ability, conferring to RNA molecules a relevant role in FUS-ALS pathogenesis [69]. 

Although much emphasis has been placed on the influence that these ALS causative genes exert on the metabolism of protein-coding RNAs, it is time to complete the biological context of the disease by highlighting the contribution of different classes of ncRNAs with regulatory activities.

Notably, a recent transcriptome profiling of both coding and long noncoding RNAs (lncRNAs) in peripheral blood mononuclear cells of unmutated sALS patients [70] versus healthy controls highlighted that the majority of differentially expressed genes belong to the nonprotein-coding class. In particular, among the 380 differentially expressed genes, 293 were lncRNAs (183 upregulated and 110 downregulated genes) whereas 87 were mRNAs (30 downregulated and 57 upregulated) [71]. It is noteworthy that the high levels of altered noncoding transcripts were not observed in other neurodegenerations such as Alzheimer’s and Parkinson’s disease [71], which supports the hypothesis of a major involvement of the transcriptional machinery in ALS. 

## 5. Noncoding RNA Landscape 

**Figure 1:**
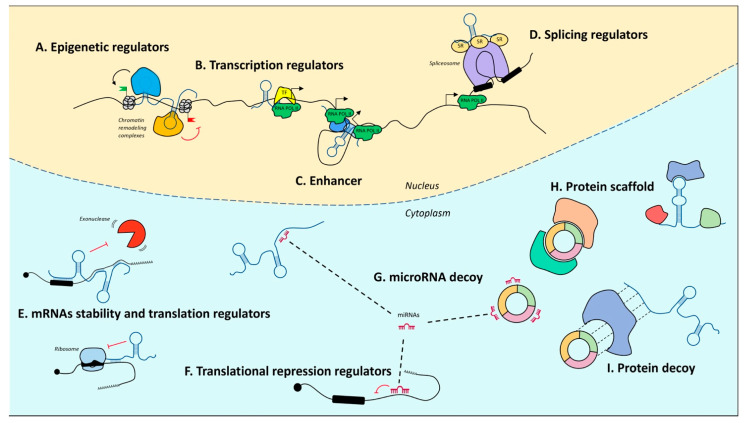
Mechanisms of action of noncoding RNAs. In the nucleus, lncRNAs can regulate gene expression by guiding epigenetic machineries (**A**), recruiting transcription factors to specific loci (**B**), acting as enhancers to promote transcription (**C**), or by recruiting splicing factors (**D**). In the cytoplasm, lncRNAs modulate mRNA stability and translation (**E**). MicroRNAs act as translational repressors (**F**) and may be sponged by both lncRNAs and circRNAs (**G**). LncRNAs and circRNAs may also act protein scaffolds (**H**) or decoys (**I**). T arrows indicate inhibition activity.

Upon the completion of the Human Genome Project, it was realized that of the three billion bases of the human genome, only approximately 2% encode proteins, whereas the most conspicuous portion produces a huge number of so-called ncRNAs [72,73,74]. Notably, their denomination refers to what they are not. In fact, with only some exceptions, they are not endowed with a codogenic potential, having only short open reading frames often interrupted by stop codons. NcRNAs are very diversified, they can be of various sizes, short (less than 200 nt) or long (greater than 200 nt), and have different conformations, both linear and circular (Figure 1). The unifying theme for all these RNAs is their function as fine regulators of gene expression, which eventually orchestrate differentiation and developmental programs through the interaction with other biological macromolecules. Moreover, their high enrichment in the nervous system (NS) led to a tremendous interest in decrypting their role in NS development and function. 

### 5.1. MicroRNAs

MiRNAs are tiny molecules of about 21–23 nt, with an established role as major posttranscriptional regulators of gene expression. Remarkably, the diversity of the miRNA repertoire increases with the organismal complexity, suggesting a role in progressively sophisticated regulation of gene expression underpinning biological complexity. Exploiting a very simple strategy, the Watson–Crick base pairing with their target mRNAs, they can inhibit protein synthesis by inducing mRNA destabilization or repressing mRNA translation (Figure 1F) [75]. Notably, a single miRNA may act pleiotropically by simultaneously regulating multiple transcripts. This property is particularly effective in canalizing the regulatory programs underlying biological processes such as apoptosis, proliferation, differentiation, and maintenance of cell identity. On the other hand, their ability to act in a combinatorial manner on the same gene makes their nature as fine regulators of gene expression much more robust, which produces only a subtle reduction in protein levels (less than 2-fold) [76]. 

Remarkably, more than half of protein-coding genes are thought to be regulated by miRNAs [77]; however, depending on the cellular context, different gene repertoires may be controlled by the same miRNA. It has been found that about 50% of the expressed miRNAs are cell-type enriched, 25% are broadly expressed, and the remaining 25% display low levels of expression regardless of cell type [78,79]. The NS is the richest source of miRNAs and their expression is highly specific for brain regions, cell types, and developmental stages. 

It is well established that miRNAs are essential for neural differentiation as well as for the maintenance of neural cell identity. In particular, they contribute to determining differentiation stage transition, by repressing leaky transcripts specific to the previous stage, and to maintaining the cell fate decision, by limiting the protein levels in the range preserving cell identity [76,80]. As an example, neuronal fate determination is heavily established by two brain-specific miRNAs, miR-9 and miR-124. They exert a wide control of the gene expression landscape by regulating chromatin remodeling complexes, repressing global inhibitors of neuronal transcription programs, and intervening in the switch to neuron-specific alternative splicing programs [81].

#### 5.1.1. MiRNA Biosynthesis Is Affected in ALS 

A complex interplay intervenes between miRNAs and miRNA biogenetic factors involved in ALS. Data indicate that the biosynthesis of miRNAs is affected in fALS and sALS cases, leading to an overall decrease in miRNA steady-state levels [82,83,84]. Other studies suggest, instead, that only specific subgroups of miRNAs are downregulated in ALS, for example, during ALS MN progenitor differentiation [85], or are affected by ALS-linked factors such as FUS and TDP-43 [86].

Along this line, a solid paradigm in the field postulates that the ALS-associated proteins FUS and TDP-43 contribute to miRNA physiological biogenesis as components of Drosha and Dicer/miRNA processing machineries [18,19]. TDP-43 promotes the maturation of a subset of miRNAs by interacting in the nucleus both with Drosha and specific miRNA primary transcripts, and in the cytoplasm with precursor miRNA terminal loops, favoring their processing by Dicer complex [58,59,60,62]. It was also demonstrated that, by regulating the abundance of the miRNA processing machinery (Microprocessor complex), TDP-43 controls the entire miRNA repertoire in in vitro differentiating neurons [55].

TDP-43 also affects the activity of miRNAs. The wild-type version of the protein or an ALS-like mutant lacking the nuclear localization signal have been demonstrated to differentially bind mature miRNAs and alter their levels in nonneuronal cell lines [87]. Making a further step towards the pathology, it was also shown that the ALS mutant TDP-43 (such as the M337V variant) is able to sequester dozens of miRNAs in cytoplasmic inclusions of mouse neuroblastoma cells [88].

Analogous crosstalk occurs between miRNAs and FUS. This protein normally enhances the production of miRNA subsets by promoting Drosha co-transcriptional recruitment on chromatin sites and by binding the corresponding miRNA primary transcripts [89]. Additionally, FUS can regulate miRNA gene silencing activity through interactions with miRNA-induced silencing complex (miRISC) components, miRNAs, and mRNA targets [90].

**Figure 2:**
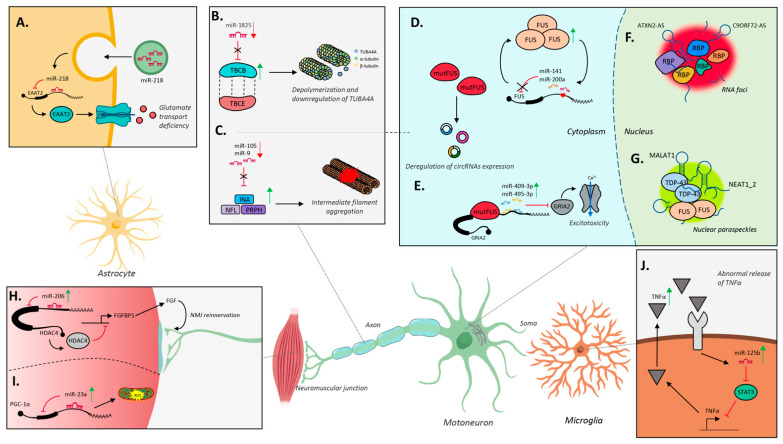
Schematic representation of noncoding RNA dysregulation examples in ALS. Green, red and T arrows indicate upregulation, downregulation and inhibition activities, respectively. (**A**) miR-218 released from degenerating MNs promotes astrocytosis in ALS; (**B**) miR-1825 dysregulation causes TUBA4A depolymerization and motor axon defects in ALS; (**C**) miR-105 and miR-9 dysregulation affects neurofilament aggregation in ALS; (**D**) left: wt FUS protein is involved in a feed-forward regulatory loop along with miR-141/200a (the red spark points to the 3′UTR *FUS* gene mutation G48A); right: mutant FUS (P525L) strongly affects circRNA biogenesis; (**E**) Mutant FUS and upregulated miR-409-3p and miR-495-3p downregulate GRIA2, causing excitotoxicity; (**F**) lncRNAs ATXN2-AS and C9ORF72-AS accumulate in ALS-associated neuronal RNA foci; (**G**) lncRNAs MALAT1 and NEAT1_2 are bound by TDP-43 and FUS proteins in nuclear paraspeckles, which are hyper-assembled in ALS; (**H**) myomiR-206 is upregulated in ALS muscle contributing to NMJ reinnervation and regeneration; (**I**) miR-23a contributes to mitochondrial dysfunction in ALS skeletal muscle; (**J**) miR-125b induces over-activation of microglia and neuroinflammation, through the STAT3 pathway.

On the other hand, some specific miRNAs have been shown to regulate *TARDBP* and *FUS*. TDP-43 is recruited in a regulatory negative feedback network with miR-181c-5p and miR-27b-3p, which is dependent on its nuclear localization. Cellular stress, which induces a redistribution of TDP-43 in the cytoplasm, correlates with the reduced production of the two miRNAs in cultured cell lines [91]. Furthermore, miR-194 and miR-b2122, which are downregulated in sALS patients, posttranscriptionally regulate both *TDP-43* and *FUS* expression. This regulatory process is disturbed in ALS, where miR-b2122 downregulation leads to an increase in FUS protein levels. Conversely, ALS-associated mutation in the *FUS 3′UTR* ablates the miR-b2122 regulatory ability [92]. These observations match previous conclusions reached by Dini Modigliani et al. [93] who showed that *FUS* 3′UTR mutations found in ALS patients caused increased protein levels and mapped to miR-141 and miR-200a binding sites. They demonstrated a feed-forward regulatory loop in which FUS induces the expression of miR-141/200a, which in turn affects protein synthesis (Figure 2D).

Overall, these and other data [94] indicate the mechanistic connection between RBPs, mutated and delocalized to SGs in ALS, and the pathological impairment of miRNA function, providing evidence for altered miRNA biogenesis/activity and regulatory circuitries as possible pathological processes in ALS.

#### 5.1.2. Integrative miRNA-Omics Studies in ALS 

Bioinformatics analyses and computational studies highlighted the importance of unraveling altered gene pathways in ALS [95,96,97,98,99]. Concordantly, several efforts were made to match and functionally link miRNAs and their target genes through integrative approaches in ALS-associated model systems. 

In this context, the first studies had a spatial connotation. Rotem et al. released the first combined inclusive profile of mRNA and miRNA expression between somatic and axonal compartments in cultured SC neurons from two in vitro ALS models, SOD1^G93A^ and TDP43^A315T^ [100]. A focus on motor axonal defects in vivo also came from Helferich et al. [101] who observed downregulation of miRNA-1825 in CNS and non-CNS organs of sALS and fALS patients (Figure 2B). Combined proteomic analyses revealed that reduction in miRNA-1825 caused the translational upregulation of tubulin folding cofactor B (*TBCB*) and the degradation of the ALS gene tubulin alpha 4a (*TUBA4A)*. Again, a whole transcriptome profiling, combined with subcellular fractionation analysis of NSC-34 human *SOD1* cells, revealed that miR-18b-5p heads a complex gene pathway made up of Hif1α, Mef2c, miR-206, Mctp1, and Rarb, with a downstream effect on cell apoptosis [102]. A very original contribution recently came from Freischmidt and colleagues [103] who identified, by proteomic and biochemical studies, the members of the fragile X protein family as interactors of a short sequence motif, enriched in a signature of previously identified ALS-related miRNAs [104,105].

Two deep mechanistic studies based on extensive RNA sequencing were performed by De Santis et al. and by Capauto et al. In human MNs derived from mutant FUS-induced pluripotent stem cells (iPSCs), De Santis and collaborators showed the decreased levels of the MN protective miRNA-375, leading to the upregulation of ELAVL4 [106], an RBP implicated in neural function and degeneration [107], and of proapoptotic targets such as p53. In concordance with this result, in the sALS wobbler mouse—a model displaying almost all clinical hallmarks of human ALS patients [108]—miRNA-375-3p downregulation resulted in inefficient p53 inhibition, increased production of reactive oxygen species, and induced apoptosis [109].

Instead, in the FUS mutant MNs differentiated from mouse embryonic stem cells (ESCs), Capauto and colleagues demonstrated the upregulation of miR-409-3p and miR-495-3p and concomitant downregulation of Gria2, a subunit of the AMPA receptor triggering a cascade of MN-damaging excitotoxic events (Figure 2E and [110]). 

With the same rationale, whole transcriptomics was performed in SC ventral horns of post mortem sALS human donors, revealing the downregulation of neuronal genes and the upregulation of glial ones [111]. Even if no miRNA/mRNA anticorrelation was highlighted in this study, it revealed strong deregulation of the SNAP25 and STX1B tSNARE proteins, involved in vesicle trafficking and Ca^2+^ dynamics. These findings once more focus on Ca^2+^ elevation and glutamate excitotoxicity as an ALS causative mechanism.

#### 5.1.3. MiR-9 and miR-124 in ALS MNs

A relevant number of studies aimed to clarify the role of specific miRNAs in ALS. In this section, we will provide an overview of the miRNAs involved in MN degenerative mechanisms, from the endoplasmic reticulum (ER) and oxidative stress to axonal transport disruption and cytoskeleton and mitochondrial defects. A significant amount of data has come from SOD1^G93A^ transgenic mice—one of the golden standards of ALS mouse models reproducing the pathological phenotype consisting in rapid degeneration of MNs [112]—or from SOD1-linked cellular models.

The deranged activity of the neural miR-9 and miR-124 has been clearly linked to neurodegeneration in ALS. In 2013, the reduced expression of miR-9 was identified as a cell-specific phenotype downstream of TDP-43 mutation in neurons derived from iPSCs [61]. A similar phenotype was confirmed in *Drosophila* [113]. Contrarily, miR-9 was found to be upregulated in the ventral horn of grey matter from SCs of *SOD1^G93A^* mice [114]. In the same year, Campos-Melo and colleagues [82] found 256 dysregulated miRNAs in sALS SCs. They compiled a panel of miRNAs—among which they validated miR-146a, miR-524-5p, and miR-582-3p—targeting the low molecular weight neurofilament gene *NEFL*, which participates in the formation of pathological cytoplasmic inclusions and was known to be decreased in sALS [115]. More miRNAs were implicated in this mechanism [116]. Recently, the same group demonstrated that the homeostasis of neurofilaments is also guaranteed by miR-9 and miR-105 (Figure 2C) by regulating genes as *NEFL*, *PRPH*, and *INA.* They encode for cytoskeleton components whose proper stoichiometry ensures neuronal cell structure and health [117]. Finally, the heavy neurofilament gene *NEFH* was also regulated by miR-9 in the context of MN diseases [118].

Besides the balance of structural proteins, miR-9 deregulation was also associated with other neuronal pathophysiological pathways in ALS. For instance, miR-9-5p was shown to be responsible for the significant decrease in expression of the P21-activated kinase *PAK4* in cell and mouse ALS models. Silencing of *PAK4* enhanced MN apoptosis through the inhibition of cyclic AMP-responsive element-binding protein 1 (CREB)-mediated neuroprotection signaling. *PAK4* overexpression in the spinal neurons of *SOD1^G93A^* mice promoted the CREB pathway, thus suppressing MN degeneration and prolonging their survival [119].

Another miRNA, miR-124, was found to be upregulated in the SC and BS of symptomatic *SOD1* mice and downregulated in neural stem cells, suggesting its defective differentiative function in ALS [120]. In the same report, a role in astrocyte differentiation of ALS mice was ascribed to miR-124, which regulates the levels of SOX2 and SOX9 transcription factors (TFs). This finding points to the relevance of noncell-autonomous phenomena in MN degeneration. Still, in the field of cell differentiation, miR-124, miR-9, and other neural miRNAs were found to be differentially expressed in *SOD1* mutant ependymal stem progenitor cells, usually quiescent in the SC but reactivated by neurodegeneration through a neurogenetic restorative mechanism [121].

Instead, in mature primary MNs, miR-124 was found to regulate the intermediate filament vimentin (*Vim*), known to physically interact with mitochondria, controlling their morphology, position, and activity [111,122]. In MNs, the miR-124/Vim axis was shown to regulate the axonal transport of mitochondria, their localization, and function [123]. Very recently, the deregulation of miR-124 has also been shown in an MN-like cell line overexpressing wt or mutant human *SOD1*. Its ectopic normalization prevented the dysregulation of several cellular phenotypes, such as neurite network, mitochondria dynamics, axonal transport, and synaptic signaling [124]. 

#### 5.1.4. Other miRNAs in ALS MNs

The expression of other miRNAs was found to be altered in *SOD1^G93A^* mice [125] and it was associated with programmed cell death, mainly via cellular stress. MiR-29 takes part in the ER stress driven by the accumulation of misfolded proteins. In ALS mouse SCs, miR-29a is upregulated by the ER stress-induced TF ATF4 [126], causing the downregulation of the antiapoptotic factor Mcl-1 [127]. In the wobbler mouse, it was reported that miR-29b-3p overexpression downregulated the proapoptotic factors BAK, BAX, and BMF, leading to apoptosis and, thus, to neurodegeneration [128]. Li et al. [129] demonstrated, instead, that the downregulation of miR-193b-3p—reported in sALS patients as well [130]—promoted cell death in the ALS *SOD1^G93A^* mouse. This was achieved by targeting tuberous sclerosis 1 (TSC1), which controls rapamycin complex 1 (mTORC1), a regulator of autophagy [131] and a neuroprotector [132]. Additionally, the zinc transporter SLC30A3, playing a protective role against ER [133] and oxidative stress [134], decreased in ALS patient SCs, as a target of the upregulated miR-5572 [135]. On the contrary, the stress sensor and neuronal protector miR-183-5p is reduced in ALS patients and mouse models, increasing the levels of the regulators of necroptosis RIPK3 and apoptosis PDCD4 [136]. Additionally, miR-335-5p was downregulated in ALS patient sera. Inhibition of miR-335-5p in neuronal cells caused an abnormal mitochondrial morphology and an increase in: (i) reactive oxygen species, (ii) superoxide dismutase activity, and (iii) apoptosis and mitophagy, suggesting a role in the MN loss [137].

Besides contributing to cellular stress, miRNAs are proposed to participate in establishing the selectivity of MN degeneration. Limb-innervating lateral motor column MNs are particularly vulnerable to degeneration and are among the first subtypes affected in ALS [138]. Such preferential susceptibility was associated with reduced expression of the miR-17∼92 cluster, accompanied by the toxic accumulation of PTEN phosphatase in spinal MN nuclei of presymptomatic *SOD1^G93A^* mice.

Finally, metabolic dysfunction is also a hallmark of age-related neurodegenerative diseases, including ALS. The aberrant accumulation of glycogen, the energy reserve of CNS, favors pathological processes and neurodegeneration in *SOD1^G93A^* mice. The regional increase in glycogen in the SC, but not in the MCx of ALS mice, was caused by defective glycogenolysis triggered by decreased levels of the brain-specific glycogen phosphorylase (PYGB). This is directly targeted by miR-338-3p, whose expression is elevated in the SC of *SOD1^G93A^* mice [139]. The latter data corroborate the long-standing idea that the central metabolism impacts MN degeneration onset or progression [140], and indicate that miRNAs may be related to the metabolic implications of the pathology. These two issues could be intriguingly linked through nutrition. The largely sporadic nature of most ALS cases indicates that extrinsic factors, including diet, may play a relevant role in ALS, representing either a potential risk or a neuroprotective factor [141]. Growing evidence demonstrates that specific nutritional regimens [142] or dietary components [143] may influence the state of ALS patients or mouse models, by affecting at several levels (microbiome, mitochondrial activity, etc.) the pathological condition of high oxidative stress. In addition, the influence of feeding on miRNA expression is a well-known biological paradigm [144]. Thus, the combination of these overall remarks suggests that the still unclear relationship among diet, miRNAs, and ALS deserves deep investigation.

#### 5.1.5. ALS miRNAs in Non-MN Cells: Microglia

Neurons undertake physiological interactions with different cell populations, such as microglia (MG), astrocytes, and oligodendrocytes. Therefore, it is not surprising that altered communication between these cytotypes characterizes the progression of neurodegenerative disorders, including ALS.

MG, the resident immune cells in the brain, exist in a homeostatic condition (resting state) that can rapidly switch to an activated, phagocytic state by sensing stimulating agents such as pathological molecules. Activated MG exert different functions corresponding to alternate phenotypes, such as the M1 (inflammatory) and M2 subtypes (proregenerative), depending on the stimulus and its intensity [145]. Two miRNAs have been studied in MG-dependent neuroinflammation in ALS, namely miR-125 and miR-155. First, Marcuzzo et al. [146] found altered levels of miR-125 along with other neural miRNAs, such as miR-9 and miR-124a, in several brain areas of late-stage diseased mice compared to age-matched controls. Then, the role of miR-125b was also analyzed in non-neuronal tissues. By miRNA transcriptional profiling, the upregulation of the immune-enriched miR-22, miR-155, miR-125b, and miR-146b was revealed in ALS MG [147]. The authors demonstrated the establishment of an aberrant regulatory network between miR-125b and the STAT3 pathway, culminating in the abnormal release of the cytokine tumor necrosis factor α (TNFα) and the overactivation of MG (Figure 2J). Later on, they also showed an additional interplay between miR-125b and the regulatory ubiquitin-editing enzyme A20, reinforcing and extending the activation of the inflammatory NF-κB pathway [148]. It is likely that more complex interactions between proinflammatory and anti-inflammatory factors under the control of miR-125b may take place in ALS MG [148].

Additionally, miR-155 is implicated in the neuroinflammation process affecting disease progression in ALS tissues, by triggering proinflammatory signaling and suppressing anti-inflammatory mediators. MiR-155 accumulates in ALS mouse and patient SCs, where a concomitant loss of the MG molecular signatures was registered [149]. Furthermore, MG overexpressing human *SOD1^G93A^* release exosomes enriched for miR-155 and miR-146 [150]. Consistent with these observations, miR-155 genetic ablation or downregulation, by antisense LNA or ASO administration, extended lifespan and disease duration in the SOD1 mouse model [151,152]. The overall scenario of neuroinflammation is even more complex. Besides miR-125 and miR-155, other “inflamma-miRs” were found to be altered in MG of presymptomatic and symptomatic *SOD1^G93A^* murine models [153]. In addition, the proinflammatory phenotype of primary and activated MG cells can be modulated by other cell types, such as mesenchymal stem cells, through the release of miRNA-containing extracellular vesicles [154]. 

#### 5.1.6. ALS miRNAs in Non-MN Cells: Astrocytes

ALS is also characterized by an increase in reactive astrocytes associated with pathological features, such as low efficiency of toxic excitatory glutamate clearance and impairment of neurotrophic factor secretion [155]. Noncell-autonomous, glial-based mechanisms of MN degeneration can be partially ascribed to miRNAs. MiR-494-3p dysregulation was detected in secreted extracellular vesicles of *C9ORF72* astrocytes and was identified as a regulator of Semaphorin 3A (*SEMA3A*) involved in axonal maintenance and MN survival [156]. Furthermore, Hoye et al. demonstrated that miR-218 could be released extracellularly by dying MNs and taken up by astrocytes. MiR-218 downregulates the expression of the glutamate reuptake transporter excitatory amino acid transporter 2 (*EAAT2*), important for the proper regulation of excitatory glutamatergic neurotransmission (Figure 2A). Consistently, miR-218 downregulation improves astrocytosis in ALS [157]. More recently, the role of miR-218 was also assessed in MNs. In ALS SC tissues, reduced miR-218 levels induced the expression of its newly identified target, the potassium channel Kv10.1 that controls neuronal activity. Furthermore, rare variants in the human miR-218-2 sequence were identified in ALS patients, which fail to undergo proper processing and are defective in neuron activity regulation [158].

Additionally, miR-146a downregulation in murine SOD1 astrocytes contributes to inflammation by impacting the TLR/NF-κB signaling pathways [159]. Interestingly, recovery of normal miR-146a levels in *SOD1* mouse cortical astrocytes was shown to mitigate their aberrant phenotype and their deleterious consequences in MNs and MG [160].

Finally, growing evidence suggests that neurovascular contributions to ALS should be considered. Loss-of-function mutations in the angiogenic factor angiogenin (*ANG1*) segregate ALS patients [161]. Deregulation of the miR-126/ANG1 axis and vascular regression, preceding MN loss, was highlighted in FUS (1–359) transgenic mice which carry a truncated version of FUS lacking the nuclear localization signal [162].

#### 5.1.7. MyomiRs in ALS

Skeletal muscle atrophy is a primary symptomatic manifestation in ALS patients. This, combined with the reciprocal and bidirectional interplay between MNs and muscle fibers at the level of the neuromuscular junction (NMJ), suggests that: (i) altered muscle structure/function may affect the onset and progression of ALS, and (ii) muscle miRNAs must be taken into account as modulators of the pathology progression. Particular attention was devoted to “myomiRs”, a subset of miRNAs enriched or specifically expressed in the skeletal muscle. They were identified and characterized as crucially implicated in the molecular network regulating muscle differentiation and regeneration by targeting myogenic TFs [163].

Together with miR-1 and miR-133, miR-206 represents the best-characterized myogenic miRNA, and one of the most studied in ALS. In their seminal paper, Williams and co-workers demonstrated that in *SOD1^G93A^* mouse muscles, miR-206 is upregulated along with miR-23a and miR-23b, whereas miR-133a, miR-133b, and miR-1 are downregulated [164]. In these myofibers, miR-206 exerts a protective function for tissue and NMJ regeneration. Indeed, it is induced by the myogenic TFs MyoD and myogenin in response to skeletal muscle denervation at the onset of neurological symptoms. MiR-206 acts by suppressing the levels of the muscular HDAC4 protein, which in turn promotes NMJ reinnervation and regeneration by inducing the expression of the fibroblast growth factor-binding protein-1 (*FGFBP1*, Figure 2H).

Further studies confirmed the consistency of miR-206 in the ALS contexts [165,166], extending to patients the previous observations underlying its relevance and characterizing the pathogenic role of additional myomiRs. Bruneteau and colleagues tried to investigate the prognostic value of the miR-206/HDAC4 pathway, highlighting an increasing trend of miR-206 expression in long-term survivor patients [167]. Additional validation of this regulatory module was provided more recently [168]. Interestingly, an increase in miR-206 was found in the CSF and the plasma of patients subjected to a potential therapeutic treatment through autologous administration of early hematopoietic cells [169].

Instead, Russell and collaborators revealed the upregulation of other miRNAs in ALS skeletal muscle, such as miR-23a, miR-29b, miR-31, and miR-455 [170]. MiR-23a was proposed as a direct regulator of *PGC-1α* expression (Figure 2I), whose signaling networks are involved in mitochondrial biogenesis and function [171]. Other factors, related to mitochondrial fusion and electron transport chain activity, were demonstrated to be downregulated in transgenic mice overexpressing miR-23a, suggesting an explanation for the mitochondrial dysfunction observed in the skeletal muscle of ALS patients. 

By analyzing a time-course of skeletal muscle biopsies from ALS patients, Jensen and co-workers [172] demonstrated that miR-1, miR-26a, miR-133a, and miR-455 were reduced in ALS patients, suggesting an alteration of both cell proliferation and differentiation. However, this specific role of myomiRs in ALS has been poorly investigated [173].

Finally, several studies pointed to the expression of myomiRs for patient stratification in terms of disease progression or duration and age of the onset [174,175,176]. Overall, these reports indicate the centrality of myomiRs in ALS and, more generally, in muscular atrophy-causing disorders [177]. Additionally, we must consider that the implication of the muscular component in ALS is even more complex than described, also considering that miRNAs not strictly referred to as myogenic can participate in muscle-mediated, pathogenic mechanisms. An example comes from miR-126-5p, whose reduced levels were reported in presymptomatic ALS mouse models, along with an increase in its targets, the axon-destabilizing *SEMA3*, and its co-receptor neuropilin [178].

The involvement of muscular miRNAs in ALS, besides representing a biological facet of the disease, may also have a practical connotation in the management of the pathology. It is well assessed that miRNA expression responds to exercise [179]. On the other hand, therapeutic training appears to be beneficial to ALS patients’ health [180]. On these bases, emphasis is placed on establishing whether these two issues are linked. Pioneering studies correlating clinical scales with circulating miRNA expression have detected lower levels of myomiRs in serum of ALS patients after aerobic exercise [181]. This finding suggests that myomiRs could represent suitable ALS biomarkers (Section 5.4) to evaluate both the disease progression and the response to physical rehabilitation and skeletal muscle recovery.

### 5.2. Long Noncoding RNAs

Similarly to protein-coding genes, it was estimated that about 2% of the human genome is transcribed into lncRNAs [182]. They are longer than 200 nt and share several features with mRNAs, being mainly transcribed by RNA polymerase II, capped, spliced, and polyadenylated. Otherwise, the majority of lncRNAs are not evolutionarily conserved at the sequence level [183,184], are expressed at lower levels, and exhibit higher tissue-specific expression patterns compared to mRNAs [185]. The main property underlying lncRNA functional versatility is their ability to fold into diverse secondary structures—such as stems, loops, and hairpins—and into complex three-dimensional structures that are key to determining their interaction and, therefore, their biological activity [186]. Through the ability to establish specific interactions with nucleic acids—mRNA, miRNA, DNA—and with proteins ([187] and Figure 1), lncRNAs act as crucial regulators of gene expression in several biological processes. In particular, they may act at the epigenetic (Figure 1A), transcriptional (Figure 1B,C), or posttranscriptional levels (Figure 1D,E) and exert their function *in cis* or *in trans* by recruiting, assembling, modifying, or scaffolding other macromolecules ([188] and Figure 1G–I). Moreover, their function is also determined by their subcellular localization that can be nuclear, cytoplasmic, or both (Figure 1).

With regard to the NS, a striking 40% (corresponding to 4000–20,000 lncRNA genes) of lncRNAs are expressed specifically in the brain, where their expression is exceptionally cell-, region-, and tissue-specific [189,190,191]. This expression pattern provides them with the ability to mark subpopulations of neural and neuronal cell types better than protein-coding genes during cortical development [192]. Compared to lncRNAs expressed in other tissues, the brain-specific lncRNAs display the highest evolutionary conservation, both in terms of sequence and structure, and display a preferential location in close proximity to protein-coding genes that are active in NS development and transcriptional regulation [193]. The findings that their expression is highly regulated during brain development and in response to neuronal activity argue for their involvement in NS structure and function. Indeed, they participate in all the stages along the differentiation process from pluripotent to postmitotic cells. Examples are the lncRNAs RMST and HOTAIRM1. The first one, during in vitro neural differentiation, drives the recruitment of the TF SOX2 that in turn activates neurogenesis-promoting genes, such as *DLX1*, *ASCL1*, *HEY2*, and *SPS* [194]. The lncRNA HOTAIRM1 represses the expression of the master gene Neurogenin 2 in the transition from neuronal precursors to neuronal cells, ensuring the correct timing of neuronal differentiation [195]. NS lncRNAs are also crucially involved in synaptogenesis, a process allowing the establishment of neuronal connections that are essential for normal brain function. Among these lncRNAs are BC1/BC200, that regulates spatially restricted synaptic turnover in vivo, and MALAT1, that regulates synaptic density in in vitro cultured hippocampal neurons. Another important process regulated by lncRNAs is neuronal plasticity that underpins learning, memory, and cognition [196]. A handful of lncRNAs have been proposed as possibly involved in ALS pathogenesis and are reported below. In most of the cases, a clear mechanistic implication in MN degeneration has not been demonstrated, even though some relevance could be attributed to antisense transcription and nucleotide expansions.

#### 5.2.1. C9ORF72-AS

From an epidemiological standpoint, *C9ORF72* is the first ALS gene. It functions in neuronal proteostasis [197] and normally carries up to 20 repeats of the hexanucleotide sequence GGGGCC in the first intron of its locus [198]. *C9ORF72* variants carrying repetition expansions ranging from hundreds to thousands represent the most common genetic cause of fALS (up to 40% of cases) and FTD (25% of patients) [44,199,200]. Both loss of normal *C9ORF72* function and gain of repeat expansion-associated toxic activity have been linked to ALS and FTD [201].

Interestingly, the *C9ORF72* genomic region was shown to be transcribed bidirectionally. In pathological conditions, the sense transcript is translated—through a non-canonical repeat-associated non-AUG (RAN) initiation event—in a six dipeptide repeat-containing protein (DRP, [48,202,203]), which accumulates in neuronal cytoplasmic aggregates along with the transcript. Similarly, the antisense RNA *C9ORF72-AS*, which contains the expanded reverse-repeated hexanucleotide (GGCCCC)n, also localizes into disease-associated nuclear RNA foci (Figure 2F and [45,203,204]) whose abundance positively correlates with the severity of ALS and FTD symptoms [199,205].

The role of *C9ORF72-AS* in MN degeneration is still debated and contradictory in comparison to the sense transcript [206,207]. However, at least two pieces of evidence point to its relevance: (1) selective *C9ORF72-AS* knockdown by ASOs attenuates, but does not fully rescue, molecular hallmarks and cellular defects in MNs carrying the expansions [46,49,208,209]; (2) antisense (but not sense) RNA foci are specifically associated with mislocalized TDP-43 in *C9ORF72* patient MNs [205,210]. An additional issue is to discriminate the contribution of the aberrant RNA or of the derived DRP to motoneuropathy [211]. This question was addressed by raising dedicated animal systems (reviewed in [212]). While “RNA-only” *Drosophila* models seem to tolerate sense and antisense RNAs [213,214,215] well, zebrafish embryos injected with RNAs consisting of dozens of sense and antisense repeats showed, in the absence of DRP, reduced axonal outgrowth and aberrant branching [216]. This is like what was observed upon *SOD1* and *TARDBP* mutations, supporting the possibility that RNA may mediate the toxicity of *C9ORF72*. Additionally, data from mouse models carrying the full-length human *C9ORF72* locus with repeat expansions are ambiguous [209,217,218,219]. Only some of them show motor or cognitive defects, probably indicating any influence of the experimental conditions or genetic backgrounds.

In conclusion, the current view proposes that *C9ORF72-AS* may mainly contribute to ALS and FTD not only by hijacking RBPs in RNA foci, but also by participating in gene expression deregulation through the formation of peculiar conformational structures [220,221].

#### 5.2.2. ATXN2-AS

The ubiquitous protein ATXN2, localized at the Golgi apparatus and the ER, regulates several cellular pathways, from mRNA processing and translation to endocytosis and energy metabolism [222]. Its mutation is widely associated with neurodegeneration. Expansions of ATXN2 polyQ repeats (from the physiological 22 copies to more than 33) cause spinocerebellar ataxia type 2 (SCA-2; [223,224]), an autosomal-dominant disorder mainly impairing cerebellar neuron circuits. Differently, a number of polyQ repeats of about 30 copies correlate with a higher risk to develop ALS [225]. Interestingly, in this condition, ATXN2 interacts with FUS and TDP-43 [225,226].

Similar to *C9ORF72*, the *ATXN2* locus is also transcribed in both directions, producing the natural antisense transcript *ATXN2-AS* [227]. In ALS lymphoblastoid lines, *ATXN2-AS* is expressed both as a normal and expanded transcript, that was shown to trigger toxicity in neuronal-like neuroblastoma cells and primary mouse cortical neurons, independently from protein translation. It has been proposed that the expansion may disrupt the function of *ATXN2-AS*, which does not seem to deal with the expression of the sense RNA. However, similarly to other expansion repeat diseases, in neurodegenerations associated with *ATXN2* transcripts, mutant RNAs may also interact with RBPs normally required for ribosomal RNA (rRNA) processing and mRNA splicing, sequestering them to aberrant RNA foci (Figure 2F and [228]).

#### 5.2.3. The Interplay between lncRNAs and ALS Genes

At least two lines of evidence demonstrate the crucial interplay occurring between lncRNAs and the pleiotropic RBPs FUS and TDP-43. On one side, a direct interaction between the proteins and RNA moieties was demonstrated by several studies. They exploited CLIP approaches (declined according to several methodological variants) followed by transcriptome profiling, or specific candidate-oriented analyses, such as biochemical purification (RNA pull down) or imaging techniques. 

On the other side, lncRNA expression was shown to be altered in proteinopathy samples or in experimental conditions where a depletion, functional inhibition, or mutation of TDP-43 and FUS intervened. All these issues have been reviewed elsewhere [229]. Below, we report some recent and specific examples of the association between lncRNAs and FUS or TDP-43 in conditions mimicking ALS.

#### 5.2.4. NEAT1

Nuclear-enriched abundant transcript 1 (expressed as *NEAT1_1* and *NEAT1_2* isoforms) is an lncRNA known to function as a chromatin regulator and, through its scaffolding activity, as an architectural organizer of subnuclear structures called paraspeckles [230]. Several IP approaches revealed the direct binding between *NEAT1* and TDP-43 or FUS [231,232,233,234]. 

By individual nucleotide resolution UV CLIP (iCLIP) in sporadic FTD cortical brain tissues containing TDP inclusions, Tollervey and colleagues revealed that TDP-43 binds *NEAT1* whose expression significantly increases in this pathology, justifying its enriched association with TDP-43. The same behavior was described for metastasis-associated lung adenocarcinoma transcript 1 (MALAT1) that recruits splicing factors to nuclear speckles and affects serine and arginine-rich (SR) protein phosphorylation (Figure 2G and [231]). Conversely, the maternally expressed lncRNA *Meg3*, relevant to MN cell fate determination [235], showed a significant downregulation in FTD-TDP and a reduction in TDP-43 binding. 

The role of *NEAT1* and paraspeckles in ALS has been addressed and it is still debated. The occurrence of paraspeckles decreased significantly upon TDP-43 or FUS knockdown in cultured cells [236], but paraspeckle hyper-assembly was observed downstream of TDP-43 loss of function in ALS [237]. By electron microscopy analysis combined with in situ hybridization, Nishimoto and colleagues demonstrated *NEAT1_2* upregulation and increased paraspeckle formation frequency during the early phases of ALS pathogenesis. This, in combination with the antiapoptotic activity of paraspeckles, suggests a compensatory mechanism to promote MN survival at the disease onset [238].

In addition, nearly all *NEAT1_2* foci overlapped endogenous TDP-43 and FUS aggregates in the nucleus of ALS MNs. The interplay between ALS-associated RBPs and *NEAT1* also affects its biogenesis, since depletion of FUS, TDP-43, or Matrin 3 leads to enhanced *NEAT1_2* expression [236,239]. Consistently, FUS mutations impair *NEAT1* transcription and paraspeckle assembly, counteracting the supposed paraspeckle-mediated mechanisms of (moto)neuron protection. In conclusion, the protective role of *NEAT1* remains unclear, since its induction in MN-like cell lines was shown to promote neurotoxicity, causing neuronal cell damage and death [240]. Conversely, the upregulation of *NEAT1_1* ameliorates TDP-43 toxicity in *Drosophila* and yeast models of TDP-43 proteinopathy [241].

#### 5.2.5. Other lncRNAs in ALS

LncRNA expression was profiled at the transcriptome level in ALS in vitro model systems or, alternatively, specific noncoding transcripts were analyzed in human or animal MNs and then verified in ALS conditions. Biscarini et al. [242] identified lncRNAs differentially expressed upon MN differentiation from mouse ESCs. Out of twelve candidates probably carrying out a function in the SC being upregulated in MNs, three transcripts (*Lhx1os*, *lncMN-1*, and *lncMN-2*) were selected for their enrichment versus the non-MN cell population and for their conservation in humans. Importantly, a deregulation of these species in mouse MNs expressing the equivalent of the severe ALS FUS mutation P525L was shown. A functional and mechanistic association with ALS is still not known for these lncRNAs. However, it is noteworthy that the protein-coding genes divergently transcribed from *Lhx1os* and *lncMN-1* loci, which are associated with MN differentiation and cell adhesion respectively, showed the same trend of deregulation as their noncoding counterparts upon FUS mutation. This suggests a co-regulated response to FUS for these bidirectional transcription units.

The *Drosophila* lncRNA *heat-shock RNA ω* (*hsrω*) is also linked to ALS-associated RBPs. On one side, TDP-43 binds the *hsrω* locus and activates its transcription [243]. Furthermore, its human orthologue satellite III repeat RNA (Sat III) shows an increased expression in FTD patient tissues and in a cellular disease model overexpressing TDP-43 [244]. On the other hand, dFUS interacts with *hsrω* whose depletion causes FUS cytoplasmic delocalization and loss of nuclear function. Finally, MN-specific knockdown of *hsrω* impairs locomotion in larval and adult flies and induces MN presynaptic defects [245]. This exemplifies how FUS and TDP-43 may potently converge on the (de)regulation of specific transcripts and exacerbate MN dysfunctions in ALS.

Lately, the expression of a panel of eight lncRNAs (*linc-Enc1*, *linc–Brn1a*, *linc–Brn1b*, *linc-p21*, *Hottip*, *Tug1*, *Eldrr*, and *Fendr*), previously characterized in mouse development and tumorigenesis, was found to be deregulated in brain and SC areas of the *SOD1^G93A^* mouse, with *linc-p21* being altered in all the tissues analyzed. Deranged levels of these lncRNAs were also detected in ALS cell model systems [246].

### 5.3. Circular RNAs 

This subclass of lncRNAs, considered as by-products of splicing errors for many years, more recently has become the object of intense studies. The majority of circular RNAs (circRNAs) derive from protein-coding genes through a non-canonical splicing event, called back-splicing, during which the downstream 5′ splice site is covalently bonded to an upstream 3′ splice site in a reversed orientation. This process, which requires spliceosomal machinery and occurs co-transcriptionally, results in a closed-loop structure that is responsible for the high stability and the accumulation of circRNAs in the cell [247]. These RNAs may consist of one or more exons or be exclusively intronic and their length can vary from 100 bp to 4 kb [248]. To add a layer of diversity, circRNA isoforms with the same junction, but different internal exons, may be released from the same gene locus [247]. They have been detected in many species, from plants to animals, are evolutionarily conserved [249], and display a tissue and developmental stage-specific expression [250]. In particular, circRNAs are significantly enriched in the brain, where 20% of protein-coding genes produce these molecules [247]. Notably, Gene Ontology analysis revealed that most of them derive from genes coding for synaptic proteins. This suggests their possible involvement in synaptic function, for instance, as scaffolds for the delivery of RNAs and proteins to the synapses [250]. Accordingly, upregulation of circRNA expression was observed during hippocampal development from stage E18 to P30, reaching the highest levels at the onset of synaptogenesis [247]. Furthermore, their expression profile was also investigated upon induction of synaptic plasticity in cultured hippocampal neurons. It showed that, unlike their linear host transcripts, the expression of a set of circRNAs was regulated by neural plasticity, with 37 being upregulated and five downregulated [247].

Their function and mechanisms of action are still poorly understood. Most evidence points to a regulatory role of gene expression carried out by their ability to act as a molecular decoy for miRNAs or RBPs, which are sequestered from their natural targets. Alternatively, circRNAs may control the transcription of their host genes [251]. Moreover, for a few of them, a role as a template for protein translation, relying on a CAP-independent mechanism, has been determined [252,253]. Importantly, modulation of their expression has been associated with neurological diseases.

#### Circular RNAs in ALS

To date, the link between circRNAs and ALS has been fragmentarily explored. The first study in the field was performed by Errichelli et al. in mouse ESC-derived MNs [254]. They highlighted a function for FUS in the processing of circRNAs, through its binding to the intronic sequences adjacent to the circularizing exons. Upon *FUS* depletion, circRNA expression was unbalanced compared to the linear counterparts, which accumulate normally. The authors also determined that FUS can directly impact the biogenesis of specific circRNAs, either positively or negatively. Finally, they suggest that circRNA biosynthesis may be affected by pathogenic FUS mutations through a mechanism possibly affecting splicing regulation (Figure 2D).

The impact of ALS-associated RBPs on the steady-state expression of circRNAs was also confirmed through the generation of a conditional mouse with a TDP-43 depletion in the forebrain, that exhibited a spectrum of FTD-like aberrant behaviors. RNA-seq data revealed that hundreds of circRNAs in the neocortex were significantly and differentially expressed between the TDP-43 KO and control mice [255].

From the mechanistic side, very recently, a study revealed that a *C9ORF72*-derived, intron-containing G-repeat can form nuclear RNA granules in vitro. It is also stabilized as a circRNA in the cytoplasm where it can function as a template for the translation of DRP, explaining how *C9ORF72* intronic expansions may contribute to ALS [256].

### 5.4. Noncoding RNAs as ALS Biomarkers 

A bulk of studies focused on miRNAs to investigate their potential role as biomarkers for accurate ALS diagnosis, prognosis prediction, or disease progression. They were based on miRNA differential and reproducible detection in human ALS samples. 

MiRNA signatures were identified by high- or low-throughput approaches (next-generation sequencing, microarray profiling, PCR arrays) from easy-to-reach ALS patient biological specimens, such as circulating body fluids, cellular fractions, or muscle biopsies. In 2012, a pioneering study by De Felice and colleagues revealed eight miRNAs, namely miR-451, miR-1275, miR-328, miR-638, miR-149, miR-665, miR-583, and miR-338-3p, that were significantly deregulated in sALS patient leukocytes. MiR-338-3p was previously found to be altered in ALS brains [257].

Later, multiple observations deepened the relationship between ALS and deregulated miRNA levels, identifying miRNA subsets that could distinguish patients from healthy subjects. To this purpose, different kinds of biological sources were employed, from blood components [258,259,260,261,262,263,264,265] up to formalin-fixed paraffin-embedded samples [266]. Due to their enriched miRNA content, their fundamental role in intercellular communication, and cargo diffusion in the surrounding environment [267], particular attention was devoted to extracellular vesicles (microvesicles and exosomes). A growing number of studies aim to identify the miRNAs transported by these carriers in normal vs. pathological cells [150,156,268], in control vs. ALS mice [154,269], or, more importantly, in ALS patients vs. healthy donors [270,271,272,273,274,275]. Finally, to match more closely the pathophysiological status of CNS, attention was also paid to the CSF as a source of data [149,276,277,278,279,280]. A comprehensive list of ALS circulating miRNAs is reported in Table 1.

Differently, several reports have profiled miRNAs from in vivo ALS models—mainly from SOD1 transgenic mice—at the symptomatic [283] or at the preclinical stage [285], followed by validations of single species in humans. In some cases, a correlation between altered miRNA expression levels and ALS functional rating scale or muscle strength emerged. 

As for prognosis and progression, a recent quantitative longitudinal analysis deserves attention. It was performed by Dobrowolny et al. [284] on selected miRNAs from patient sera during disease development. They showed that the early stage of ALS displays low levels of miR-199a-5p, miR-133a, and miR-423-3p and, conversely, high levels of miR-151a-5p and miR-206, which also predict a slower functionality decline.

In conclusion, among all the potential ALS miRNA biomarkers which are progressively emerging, those already associated with the development or physiology of neural, (moto)neuronal, and muscle cells are of major interest. Between the species overexpressed in ALS, miR-9 [281,282,286], miR-124 [279], miR-206 [165,281,282,286], miR-338-3p [257,287], and miR-133b [258] should be mentioned. Complementarily, miRNAs such as miR-132 [276], miR-128 [260], and miR-183 [130,260] were described as downregulated. Despite these major efforts, current data on miRNAs as sensors in ALS are sometimes contradictory and still not clear enough for a rapid translation into clinical routine.

By contrast, only very few studies focused on lncRNAs (either linear or circular) differentially expressed in biological samples of ALS patients. In 2018, Gagliardi and co-authors detected 293 lncRNAs that were dysregulated in peripheral blood mononuclear cells from uncharacterized sALS patients. Furthermore, 21 species were found to be altered in patients carrying a FUS mutation, 11 in TARDBP-associated cases and two in SOD1 mutant patients [70]. Most of these candidates were unknown and, at least in some cases, antisense to specific RNAs, suggesting plausible sequence complementarity-based mechanisms of action. As for circRNAs, 425 species were identified by microarray profiling as differentially expressed in leukocytes of sALS patients, and seven out of 10 selected species were validated in a larger cohort of patients [288]. Some of them revealed high statistical significance and biological relevance, based on the identity of the host gene or the presence of putative binding sites for miRNAs deregulated in ALS. Finally, Hosaka and colleagues [289] showed that extracellular RNAs, including circRNAs, are differentially edited in mice lacking ADAR2, the deaminase responsible for adenosine to inosine posttranscriptional modification of transcripts, which is downregulated in sALS [290]. If confirmed in ALS samples, this may suggest circRNA editing as a disease biomarker.

## 6. Conclusions

The multi-genetic traits of ALS make it difficult to univocally define this disease. Among the several descriptions, ALS was also labeled as an RNA disorder, which mainly derives from the alterations that its causative genes provoke in RNA metabolism. Furthermore, the involvement of ncRNAs in ALS etiopathogenesis, which has been progressively emerging over the years, represents an additional and intriguing justification to this definition. The study of ncRNAs in ALS provides at least two scientific advancements in the comprehension of the pathology. The first one is a conceptual contribution to extend and clarify the gene pathways underlying the pathology. This aspect is particularly relevant for the future development of targeted therapies. Nevertheless, the understanding of ALS is still a long way off, due to the high heterogeneity and interlacement of pathogenic mechanisms converging onto the MN degeneration. However, even if we are facing a still fragmented picture of the pathology, the participation of ncRNAs in several pathogenic processes, ranging from astrogliosis to muscle atrophy, oxidative stress, and inflammation, highlights their wide involvement in ALS.

The second advancement concerns the possible applications of ncRNAs as novel biomarkers for disease occurrence or stage of progression. In this regard, miRNAs represent the most appealing class of noncoding molecules to date. Oncological studies highlighted miRNAs as powerful clinical indicators for their high tissue specificity in health and disease and their easy detectability in body fluids as stable molecules. Even if several issues, such as heterogeneous ALS etiology and data collection or analysis, counteract miRNAs’ rapid translation into clinical routine, it is widely assumed that they may function as ALS biomarkers. They may help to diagnose the occurrence of the disease or to characterize its development through association with patients’ functional rates.

Currently, no effective treatment for ALS is available to halt or reverse the progression of the disease. Prospectively, ncRNAs are suitable for introduction in the (pre)clinical circuits as therapeutic agents. This may apply especially to miRNAs, whose gene targets are easy to predict and validate and whose sequence/structure allows easier manipulations. These features, combined with the consolidation of intervention strategies, such as miRNA functional inhibition via ASOs or miRNA functional recovery through miRNA mimics or AAV-based gene therapy, have already generated promising results. The manipulation of specific miRNAs, such as miR-155 [151,152], miR-129-5p [291], and miR-17∼92 [138], has proved to be effective for suppressing adverse phenotypes, favoring the integrity and the amount of MNs, slowing down ALS progression, and promoting the survival of affected animals. MiRNA-based treatments targeting the SOD1 pathway seem to be particularly promising. Several approaches have already been proposed, based on the use of either native or artificial miRNAs combined with potent viral vector delivery systems in transgenic *SOD1^G93A^* mice and primates [292,293,294,295,296]. Furthermore, transcripts of other ALS genes, such as *C9ORF72*, have also been targeted through miRNAs [297]. These pieces of evidence bring the prospective application of miRNA therapies to treat ALS in humans closer ([298] and https://www.neals.org/als-trials/1331).

## Figures and Tables

**Table 1:** Differentially expressed microRNAs detected in ALS biofluids.

Sample	Disease	Upregulated	Downregulated	Reference
Cortico-spinal tract tissue (EV)	C9orf72 ALS		miRNA-494-3p	[156]
Cerebro-spinal fluid	sALS, fALS	miRNA-27b, miRNA-99b, miRNA-146a, miRNA-150, miRNA-328, miRNA-532-3p		[149]
Cerebro-spinal fluid	sALS	miRNA-143-5p, miRNA-574-5p	miRNA-132-3p, miRNA-132-5p, miRNA-143-3p	[276]
Cerebro-spinal fluid	sALS	miRNA-181a-5p	let7a-5p, let7b-5p, let7f-5p, miRNA-15b-5p, miRNA-21-5p, miRNA-148-3p, miRNA-195-5p	[278]
Cerebro-spinal fluid	sALS	miRNA-9-5p, miRNA-23b-3p, miRNA-27b-3p, miRNA-99b-5p, miRNA-124-3p, miRNA-126-5p, miRNA-127-3p	let-7f-5p, miRNA-i50-5p, miRNA-142-5p, miRNA-378a-3p	[281]
Cerebro-spinal fluid	sALS	miRNA-9-5p, miRNA-27b-3p, miRNA-124-3p, miRNA-125b-2-3p, miRNA-127-3p, miRNA-143-3p	let7f-5p, miRNA-16-5p, miRNA-28-3p, miRNA-92a-5p, miRNA-142-5p, miRNA-146a-3p, miRNA-150-5p, miRNA-378a-3p, miRNA-486-5p	[279]
Peripheral blood mononuclear cells	ALS	miRNA-183, miRNA-193b, miRNA-451, miRNA-3935		[130]
Plasma	sALS	miRNA-4649-5p	miRNA-4299	[261]
Plasma	sALS	miRNA-206, miRNA-424		[262]
Plasma	ALS	miRNA-9, miRNA-129-3p, miRNA-206, miRNA-335-5p, miRNA-338-3p		[282]
Plasma	sALS		let-7a-5p, let-7d-5p, let-7f-5p, let-7g-5p, let-7i-5p, miRNA-15a-5p, miRNA-15b-5p, miRNA-16-5p, miRNA-22-3p, miRNA-23a-3p, miRNA-26a-5p, miRNA-26b-5p, miRNA-27b-3p, miRNA-28-3p, miRNA-30b-5p, miRNA-30c-5p, miRNA-93-5p, miRNA-103a-3p, miRNA-106b-3p, miRNA-128-3p, miRNA-130a-3p, miRNA-130b-3p, miRNA-144-5p, miRNA-148a-3p, miRNA-148b-3p, miRNA-151a-5p, miRNA-151b, miRNA-182-5p, miRNA-183-5p, miRNA-186-5p, miRNA-221-3p, miRNA-223-3p, miRNA-342-3p, miRNA-425-5p, miRNA-451a, miRNA-532-5p, miRNA-550a-3p, miRNA-584-5p	[260]
Plasma	ALS	miRNA-532.3p, miRNA-144-3p, miRNA-15a-5p, miRNA-363-3p, miRNA-183-5p	let-7c-5p, miRNA-4454, miRNA-9-1-5p, miRNA-9-3-5p, miRNA-338-3p, miRNA-9-2-5p, miRNA-100-5p, miRNA-7977, miRNA-1246, miRNA-664a-5p, miRNA-7641-1, miRNA-1290, miRNA-4286, miRNA-181b-1-5p, miRNA-1260b, miRNA-181b-2-5p, miRNA-127-3p, miRNA-181a-2-5p, miRNA-181a-1-5p, miRNA-199b-3p, miRNA-199a-1-3p	[271]
Plasma	C9orf72 ALS	miRNA-34a-5p, miRNA-345-5p	miRNA-200c-3p, miRNA-10a-3p	[264]
Plasma	sALS, fALS	let7f-5p, miRNA-106, miRNA-142, miRNA-143, miRNA-206, miRNA-4516	let7f-5p	[265]
Plasma (EV)	ALS	miRNA-24-3p, miRNA-149-3p, miRNA-371a-5p, miRNA-939-5p, miRNA-1207-5p, miRNA-3619-3p, miRNA-4298, miRNA-4484, miRNA-4505, miRNA-4688, miRNA-4700-5p, miRNA-4736, miRNA-4739	miRNA-150-3p, miRNA-634, miRNA-1268a, miRNA-1913, miRNA-2861, miRNA-3176, miRNA-3177-3p, miRNA-3605-5p, miRNA-3911, miRNA-3940-3p, miRNA-4507, miRNA-4508, miRNA-4646-5p, miRNA-4674, miRNA-4687-5p, miRNA-4745-5p, miRNA-4788	[272]
Plasma (EV)	ALS	miRNA-146a-5p, miRNA-151a-3p, miRNA-151a-5p, miRNA-199a-3p, miRNA-199a-5p	miRNA-10b-5p, miRNA-29b-3p, miRNA-4454	[273]
Plasma (EV)	sALS	miRNA-8089, miRNA-196a-5p, miRNA-3152-3p, miRNA-607, miRNA-3607-3p, miRNA-6825-3p, miRNA-7106-5p, miRNA-3976, miRNA-4492, miRNA-200a-3p, miRNA-205-5p, miRNA-6858-3p, miRNA-1273c, miRNA-6888.3p, miRNA-4302, miRNA-4634, miRNA-182-3p, miRNA-3160-3p, miRNA-1-3p, miRNA-200a-5p, miRNA-7704, miRNA-210-3p, miRNA-31-5p, miRNA-133a-3p, miRNA-34c-5p, miRNA-455-5p, miRNA-6842-5p, miRNA-3619-3p, miRNA-4279, miRNA-4508, miRNA-1469, miRNA-141-3p, miRNA-542-3p, miRNA-615-3p, miRNA-200c-3p, miRNA-4451, miRNA-18a-5p, miRNA-200b-3p, miRNA-184, miRNA-9-5p, miRNA-7c-5p, miRNA-6746-5p, miRNA-3195, miRNA-206, miRNA-6068	miRNA-493-3p, 409-3p, miRNA-323b-3p, miRNA-6073, miRNA-432-5p, miRNA-134-5p, miRNA-330-3p, miRNA-625-3p, miRNA-4446-3p, miRNA-148b-3p, miRNA-370-3p, miRNA-584-5p, miRNA-224-5p, miRNA-381-3p, miRNA-199a-5p, miRNA-654-3p, miRNA-335-3p, miRNA-543, miRNA-4433b-5p, miRNA-130b-5p, miRNA-4286, miRNA-382-5p	[275]
Serum	sALS		let-7b-5p, miRNA-132-3p, miRNA-132-5p, miRNA-143-3p, miRNA-143-5p	[276]
Serum	sALS	miR-338-3p		[277]
Serum	fALS		miRNA-1825, miRNA-1915-3p, miRNA-3665, miRNA-4530, miRNA-4745-5p	[104]
Serum	ALS	miRNA-106b, miRNA-206		[165]
Serum	sALS		miRNA-1234-3p, miRNA-1825	[105]
Serum	sALS	miRNA-143-3p, miRNA-206	miRNA-374b-5p	[281]
Serum	sALS	miRNA-142-3p	miRNA-1249-3p	[283]
Serum	ALS	miRNA-1, miRNA-19a-3p, miRNA-133a-3p, miRNA-133b, miRNA-192-3p, miRNA-192-5p, miRNA-142-3p, miRNA-144-5p	let-7d-3p, miRNA-139-5p, miRNA-320a, miRNA-320b, miRNA-320c, miRNA-425-5p	[258]
Serum	sALS, fALS	miRNA-133a, miRNA-206	miRNA-151a-5p, miRNA-199a-5p, miRNA-423-3p	[284]
Serum (EXO)	ALS		miRNA-27a-3p	[270]

EV = extracellular vesicle; EXO = exosome.

## Abbreviations

ALS	amyotrophic lateral sclerosis
ASO	antisense oligonucleotide
BS	brainstem
circRNA	circular RNA
CLIP	cross-linking immunoprecipitation
CNS	central nervous system
CSF	cerebrospinal fluid
DRP	dipeptide-repeat-containing protein
ER	endoplasmic reticulum
ESC	embryonic stem cell
fALS	familial ALS
FTD	frontotemporal dementia
iPSC	induced pluripotent stem cell
lncRNA	long noncoding RNA
MCx	motor cortex
MG	microglia
miRNA	microRNA
MN	motoneuron
mRNA	messenger RNA
ncRNA	noncoding RNA
Nf	neurofilament
NMJ	neuromuscular junction
NS	nervous system
pNfH	phosphorylated neurofilament heavy chain
RBP	RNA binding protein
RNP	ribonucleoprotein
sALS	sporadic ALS
SC	spinal cord
SG	stress granule
TF	transcription factor
UTR	untranslated region
